# Myocardial salvage by T2W-CMR: direct comparison to a non-destructive, high resolution, 3-dimensional ex-vivo assessment of the area at risk simultaneous with infarction

**DOI:** 10.1186/1532-429X-15-S1-O15

**Published:** 2013-01-30

**Authors:** Lowie M Van Assche, Christoph J Jensen, David Wendell, Michele Parker, Han W Kim, Raymond J Kim

**Affiliations:** 1 Cardiology, Duke University, Durham, NC, USA

## Background

The amount of myocardial salvage is a critical determinant of prognosis in acute myocardial infarction (AMI). T2W-CMR in combination with DE-CMR is thought to be a promising method that could presumably measure salvage. However, direct comparisons with the appropriate reference standard are limited. Additionally, even if a pathology reference standard is present, co-registration with in-vivo imaging is problematic. We recently validated a new ex-vivo CMR protocol against the reference standard of microspheres and TTC that provides 3D, non-destructive, high-resolution maps of the AAR simultaneously with infarction[1]. Because this map delineates both the AAR and infarction, salvage can be easily measured in a single image, rather than being calculated from 2 datasets. Additionally, the 3D map allows for direct matching of in-vivo DE to ex-vivo CMR and thereby further minimizing the potential for misregistration from in-vivo DE and more importantly in-vivo T2. Therefore, we sought to compare in-vivo calculated salvage to the directly measured salvage by ex-vivo CMR.

## Methods

12 canines underwent variable coronary occlusion of the LAD (45-75min) followed by reperfusion to create a range of myocardial salvage. CMR was performed 5-days post-AMI. T2W-CMR was performed using an optimized DIR-TSE sequence with reduced inter-echo spacing and significantly improved image homogeneity [2]. Following in-vivo imaging, the 3D ex-vivo protocol was performed delineating viable AAR (dark), infarcted AAR (bright) and normal myocardium (grey). Ex-vivo CMR slices were directly matched to in-vivo DE images. In-vivo salvage was calculated by subtracting infarct size by DE from T2-hyperintensity size by TSE and expressed as a percentage of T2-hyperintensity size. For ex-vivo CMR, salvage was directly measured on the matched slices and expressed as a percentage of the AAR.

## Results

**Figure 1:**
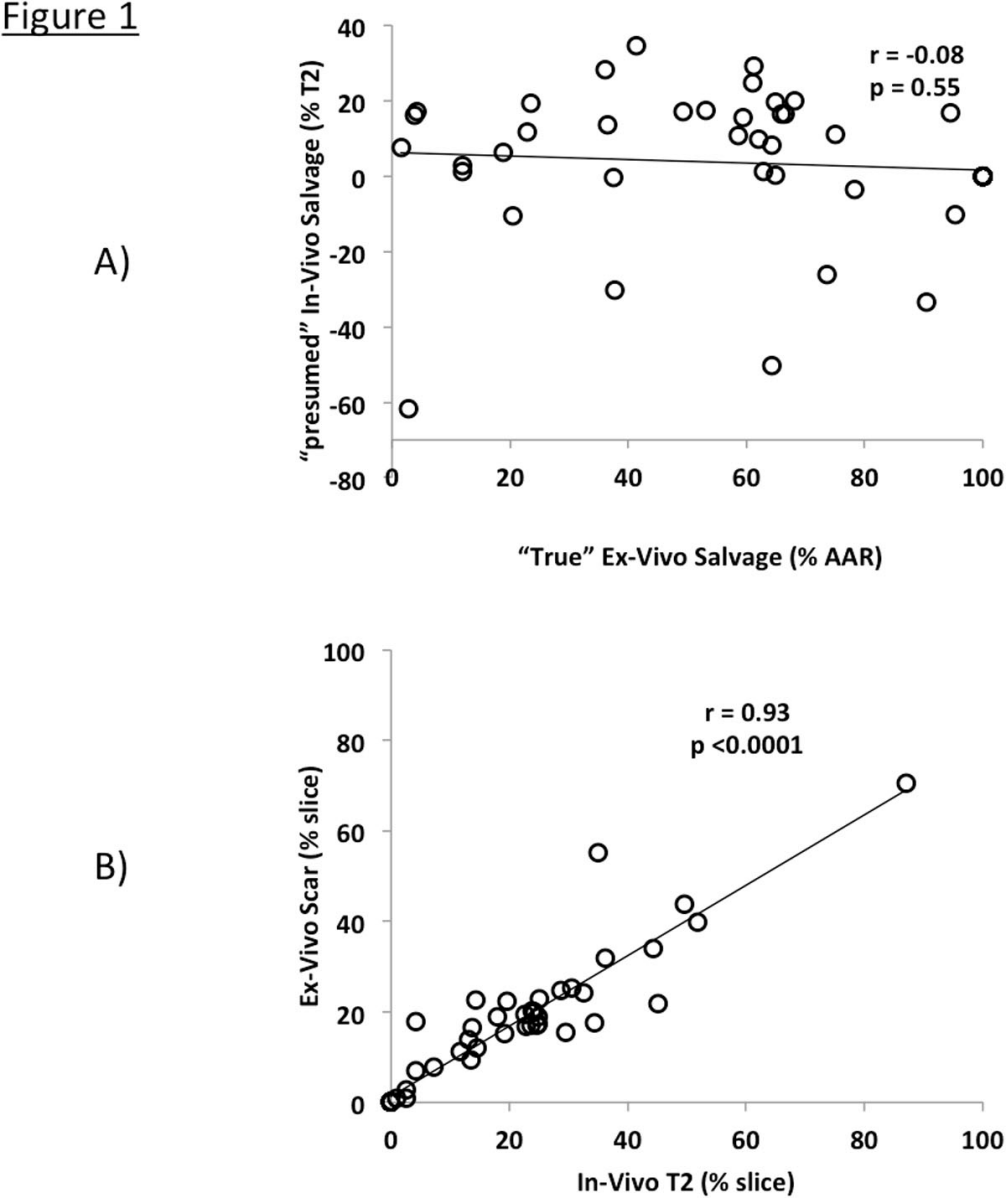


**Figure 2:**
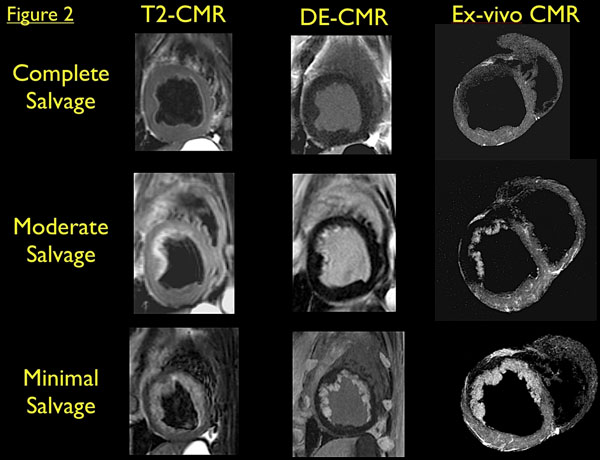


A total of 51 slices were analyzed. Mean calculated salvage by in-vivo CMR was significantly lower then mean measured salvage by ex-vivo CMR. (3.2% vs 63.6%, respectively, p<0.0001). Figure 1 shows no correlation between in-vivo and ex-vivo CMR (r=-0.08, p=0.55). Conversely, there was a significant correlation between T2 size by in-vivo CMR and scar size by the ex-vivo protocol (r=0.93, p<0.0001). Figure 2 shows examples of in-vivo T2 and DE slices compared with directly marched ex-vivo CMR.

## Conclusions

In-vivo calculated salvage did not correspond with directly measured salvage on matched ex-vivo slices. Instead, there was a strong correlation between in-vivo T2 size and ex-vivo scar size.

## Funding

Funded in part by 5R01HL064726-07.
